# Mechanisms of Visuomotor Interception

**DOI:** 10.3390/brainsci16050435

**Published:** 2026-04-22

**Authors:** Inmaculada Márquez, Mario Treviño

**Affiliations:** 1 Laboratorio de Plasticidad Cortical y Aprendizaje Perceptual, Instituto de Neurociencias, Universidad de Guadalajara, Francisco de Quevedo 180, Arcos Vallarta, Guadalajara 44130, Jalisco, Mexico; maria.marquez@academicos.udg.mx; 2 Departamento de Psicología, Centro Universitario de la Ciénega, Universidad de Guadalajara, Ocotlán 47810, Jalisco, Mexico; 3 Laboratorio de Neurofisiología, Departamento de Bioingeniería Traslacional, Centro Universitario de Ciencias Exactas e Ingenierías, Guadalajara 44430, Jalisco, Mexico

**Keywords:** visuomotor interception, predictive processing, sensorimotor control, eye movements, internal models, reinforcement learning

## Abstract

**Highlights:**

**What are the main findings?**

- Interception behavior reflects a continuous interaction between predictive processes and online feedback corrections.
- Predictive and reactive control can be quantified using geometric and temporal measures that separate spatial (“where”) and temporal (“when”) components of movement trajectories.

**What are the implications of the main findings?**

- Similar behavioral patterns can arise from different mechanisms, making them difficult to explain with a single framework.
- Combining behavioral, computational, and neural approaches is needed to identify the mechanisms of adaptive sensorimotor behavior.

**Abstract:**

Background/Objectives: Visuomotor interception requires aligning action with the future state of moving targets under sensory and motor delays. This constraint provides a tractable framework to examine how predictive and feedback-driven processes interact. This narrative review evaluates theoretical and empirical accounts of interception, with emphasis on how prediction and online control are integrated across behavioral and neural levels. Methods: We conducted a narrative synthesis of behavioral, eye-tracking, computational, and neurophysiological studies on visuomotor interception. Literature was identified through searches of PubMed, Web of Science, and Google Scholar using search terms including “visuomotor interception,” “predictive motor control,” “eye–hand coordination,” “time-to-contact,” “sensorimotor delay,” and related combinations. Studies published between 1986 and 2026 were considered, with emphasis on peer-reviewed empirical and theoretical work. Preprints were included only when directly relevant and are identified as such. The review compares internal model, ecological, and hybrid frameworks, and organizes evidence around spatial (“where”) and temporal (“when”) components of control. Results: Across paradigms, interception behavior is not well accounted for by purely predictive or reactive mechanisms. Instead, trajectories reflect a continuous interaction between anticipatory guidance and online correction. Spatial and temporal components show partial dissociation across tasks and manipulations. Available evidence supports the involvement of distributed circuits, including parietal, frontal, cerebellar, and subcortical systems, while indicating that eye movements play an active role in both information sampling and motor planning. Conclusions: Interception is best understood as the product of interacting biological, environmental, and learned constraints. Similar behavioral signatures can arise from distinct mechanisms, arguing against a unitary account. Progress requires integrating behavioral analyses with model-based and neural approaches to dissociate underlying computations.

## 1. Introduction

Natural environments contain statistical regularities that can be exploited to anticipate future events. Many sensory signals therefore function as predictive cues: their current state constrains likely future outcomes. The trajectory and speed of a moving prey or predator delimit its future position, and the early phases of an unfolding action often specify its continuation. Through experience, organisms learn these relationships, allowing behavior to be organized not only around present sensory input but also around expected future states. This capacity is essential because biological systems operate under substantial sensory and motor delays. In this narrative review, we use the term visuomotor interception to refer to goal-directed motor actions aimed at spatially and temporally coinciding with a moving target under sensory and motor delays. Our scope primarily encompasses manual interception tasks involving continuous effector control, such as joystick- or hand-guided cursor movement toward linearly moving targets, though we draw on locomotor and discrete-timing paradigms where their findings inform the discussion.

Predictive processing has been proposed as a framework to formalize how such regularities are used and how their violations are handled. In this view, the nervous system continuously generates expectations and compares them with incoming sensory input, shaping both perception and action [1,2,3]. Several theoretical frameworks address different aspects of this problem, and it is useful to distinguish the levels at which they operate. Descriptive-geometric principles, such as constant-bearing strategies and tau-based time-to-contact estimation, specify observable regularities in behavior without necessarily positing internal mechanisms. Formal control models, including optimal feedback control, internal forward/inverse models, and Bayesian inference, formalize how sensory information and prior expectations might be combined to generate motor commands. Neural implementation accounts map these computations onto specific circuits and cell types. These levels are complementary but not interchangeable; behavioral regularities consistent with a given formal model do not, by themselves, establish a particular neural implementation. This organization becomes particularly relevant in motor behavior. Visual signals require tens of milliseconds to be processed, and motor corrections take longer. By the time a response is deployed, often 150–250 ms later, the external state has already changed [4]. Under these conditions, purely reactive control is insufficient. Behavior must compensate for delay by incorporating information about the near future, particularly when the chaser lacks or does not exploit a speed advantage.

Interception tasks make this constraint explicit. Intercepting a moving target imposes a temporal ‘deadline’ under limited and delayed information. In a paradigm described previously [4], participants manipulate a joystick to guide a cursor toward a continuously moving target following piecewise linear trajectories. The setup is minimal, yet it preserves key features of natural interception: continuous motion, delayed feedback, and real-time coordination. Its advantage lies in experimental control. Task parameters can be precisely defined, which helps estimate the relative weighting of predictive and feedback-driven contributions, even if the two are rarely fully separable in practice.

The field has moved away from treating interception as either predictive or reactive. This dichotomy does not capture observed behavior. Empirical data instead support a continuum in which anticipatory and online processes coexist and vary in relative contribution depending on sensory availability and task demands [5,6]. Perception, action, and environmental structure form a coupled system rather than separable modules.

This review synthesizes current work on visuomotor interception, with an emphasis on how the authors have framed and developed the topic. Study selection was guided by thematic relevance and the authors’ domain expertise; accordingly, this is a narrative rather than a systematic review, and the coverage is not intended to be exhaustive (for reporting standards in narrative reviews, see [7]). The text is organized around two complementary perspectives. The first is biological, focusing on the constraints imposed by the effector, neural processing delays, and the distributed circuits that transform visual input into motor output. Eye movements play a central role by selecting and structuring sensory input while contributing to action planning. The second is computational. Interception can be decomposed into spatial and temporal components, estimating where and when to act, and these components do not always align. Behavioral and eye-tracking data reveal partial dissociations between them [8]. This argues against a unitary account of prediction. Interception is probably better understood as the outcome of interacting constraints, biological, environmental, and learned, implemented through task-dependent control policies. Prediction is one component of this process, but it operates alongside mechanisms that remain tightly anchored to current sensory input.

## 2. Biological Foundations of Visuomotor Interception

Visuomotor interception unfolds under multiple constraints, including the biomechanics of the effector and the finite processing speeds of neural systems. Arm and hand movements emerge from coordinated activity across multiple joints. The human hand alone comprises 27 bones, 29 joints, and more than 120 ligaments, forming a high-dimensional system capable of fine control but also introducing substantial complexity [9]. Muscles rarely act in isolation; many span multiple joints, so forces applied at one segment propagate across the limb, producing nonlinear and coupled dynamics [10]. The interface also matters. A joystick imposes mechanical constraints, such as impedance and resistance, and typically enforces a linear mapping between force and cursor displacement [8,11].

This high dimensionality introduces redundancy: multiple motor configurations can achieve the same task outcome. Rather than eliminating this redundancy, biological systems appear to exploit it. The uncontrolled manifold (UCM) framework provides one account, proposing that variability is structured to stabilize task-relevant variables, such as the distance between effector and target, despite fluctuations in underlying degrees of freedom [12,13,14,15]. Variability is therefore not simply noise but reflects selective control: dimensions that affect task success are constrained, while others remain flexible. This principle of motor abundance reframes control as the organization of variability rather than its suppression [14,16].

How this organization is implemented remains debated. Internal model theories propose that the nervous system encodes mappings between actions and their sensory consequences. Forward models use efference copies of motor commands to predict their sensory outcomes, effectively projecting the system into its immediate future [17,18,19,20]. In the canonical formulation of Wolpert et al. (1995) [19], a forward model combines the current motor command with the system’s estimated state to generate a prediction of the sensory consequences before feedback arrives. This prediction serves as a rapid internal estimate of the outcome, enabling the system to compensate for delays. The concept has since been extended beyond the original sensorimotor integration framework into broader predictive-processing and active inference formulations; however, these extensions should be distinguished from the original, more constrained proposal. Inverse models, often formalized within optimal feedback control, generate motor commands by minimizing cost functions defined over task-relevant variables such as error, variability, and energetic expenditure [20,21,22]. Adaptation studies, particularly those involving perturbations in target dynamics or visuomotor mappings, show that behavior recalibrates systematically, consistent with the updating of internal representations [17,23]. In interception, these frameworks imply continuous estimation of both target and effector states under uncertainty, enabling prospective error reduction.

An alternative account is provided by the equilibrium-point (referent configuration) hypothesis. Here, control does not rely on explicit computation of forces. Instead, the nervous system specifies desired configurations, and movement emerges from the interaction between these referent states and the intrinsic viscoelastic properties of the limb [14,15,24,25]. Multi-joint actions can thus be interpreted as trajectories of referent configurations. In interception, this view suggests that the arm–joystick system is continuously reconfigured so that its equilibrium state tracks the evolving target.

At the level of execution, additional physiological constraints shape behavior. Motor unit recruitment generally follows the size principle, enabling graded force production, though recruitment patterns depend on task demands [26]. Preparatory activity can pre-activate motor units, reducing response latency [27]. Coactivation of agonist and antagonist muscles increases limb impedance, stabilizing the system under noise and delay, but at a metabolic cost [28,29]. The degree of stability is not fixed. It is regulated dynamically, reflecting a trade-off between robustness and energy expenditure. Optimal feedback control frameworks formalize this trade-off: impedance increases when uncertainty or potential perturbations are high, and decreases when the environment is predictable [30,31]. Thus, stability is adjusted according to task demands rather than preset.

**Figure 1:**
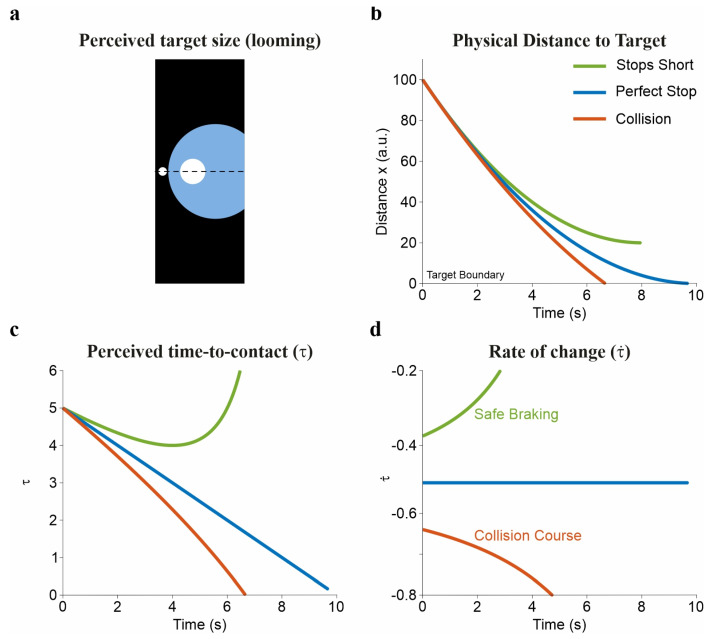
Visual and kinematic representation of time-to-contact (τ) theory during approach and braking (Conceptual illustration). The figure illustrates three constant-deceleration braking strategies toward a stationary target: safe braking where the actor stops short (green), ideal braking where the actor stops exactly at the target (blue), and hard braking resulting in collision (red). (**a**) Perceived target size (looming): the target’s retinal expansion over time, shown for the ideal braking scenario. As the actor approaches, the visual angle increases slowly at first, then expands rapidly and nonlinearly near contact. This optical expansion is the primary sensory input for estimating time-to-contact. (**b**) Physical distance to target: the physical spatial distance (*x*) between the actor and the target boundary (*x* = 0) over time. Line colors in panels (**b**–**d**) correspond to the three braking strategies (green: safe, blue: ideal, red: hard), with the color legend shown in the inset of panel (**b**). (**c**) Perceived time-to-contact (τ): the optical variable τ, defined as the inverse of the relative rate of retinal expansion, specifying the time remaining until contact if current approach velocity were maintained. (**d**) Rate of change ($\dot{\tau }$): the temporal derivative of τ, which serves as the control parameter for braking. A constant $\dot{\tau }$ value of exactly −0.5 (blue line) corresponds to ideal marginal braking. Values above −0.5 result in stopping short; values below −0.5 indicate collision. A brief description of the simulation procedures used to generate this figure is provided in the Supplemental Material.

The need to anticipate future states is also evident in perceptual mechanisms for estimating time-to-contact (TTC; Figure 1). Tau theory proposes that the ratio between an object’s visual angle to its rate of expansion determines the remaining time before contact [32,33,34]. Under looming conditions, when an object approaches the observer and its retinal projection expands, this variable can be extracted directly from the optic array without explicit computation of distance or velocity. Extensions such as τ-coupling describe how multiple gaps (e.g., between hand and target) can be coordinated through linked temporal dynamics [34].

However, the utility of τ depends on viewing geometry. It breaks down when looming information is absent, such as in lateral motion, top-down displays, or third-person perspectives. In these cases, observers rely on alternative cues, including distance–velocity relationships and geometric constraints [35,36]. Empirical evidence indicates that τ is neither necessary nor sufficient for TTC estimation. Instead, observers combine and reweight multiple sources of information depending on context [32,37]. Tau is therefore better understood as one cue among several, rather than a general solution.

Experience further shapes interception through learned regularities. Gravity provides a clear example. Targets moving under gravitational acceleration are predicted more accurately, particularly during occlusion [38]. Such effects indicate the incorporation of environmental priors derived from repeated exposure. These learned regularities can be understood within the broader framework of selection history, in which past episodes of attentional selection, shaped by reward, statistical regularities, and inter-trial priming, modulate attentional priority maps and bias sensorimotor control beyond what current goals or stimulus salience alone would predict [39]. In interception, this means that accumulated experience does not merely refine motor estimates; it also tunes attentional weighting of sensory cues and motor strategies through mechanisms that operate alongside, and sometimes independently of, deliberate top-down control.

Developmental evidence suggests that sensitivity to these regularities appears early. Infants can anticipate the reappearance of occluded objects within the first months of life, indicating early sensitivity to temporal continuity and motion structure [40]. These abilities likely reflect rapid learning interacting with basic perceptual constraints. Prediction, in this sense, is not a fixed capacity. It is a working solution used by systems that operate under delay, shaped by both immediate sensory input and accumulated experience.

## 3. Neural Circuits Supporting Visuomotor Transformation

**Figure 2:**
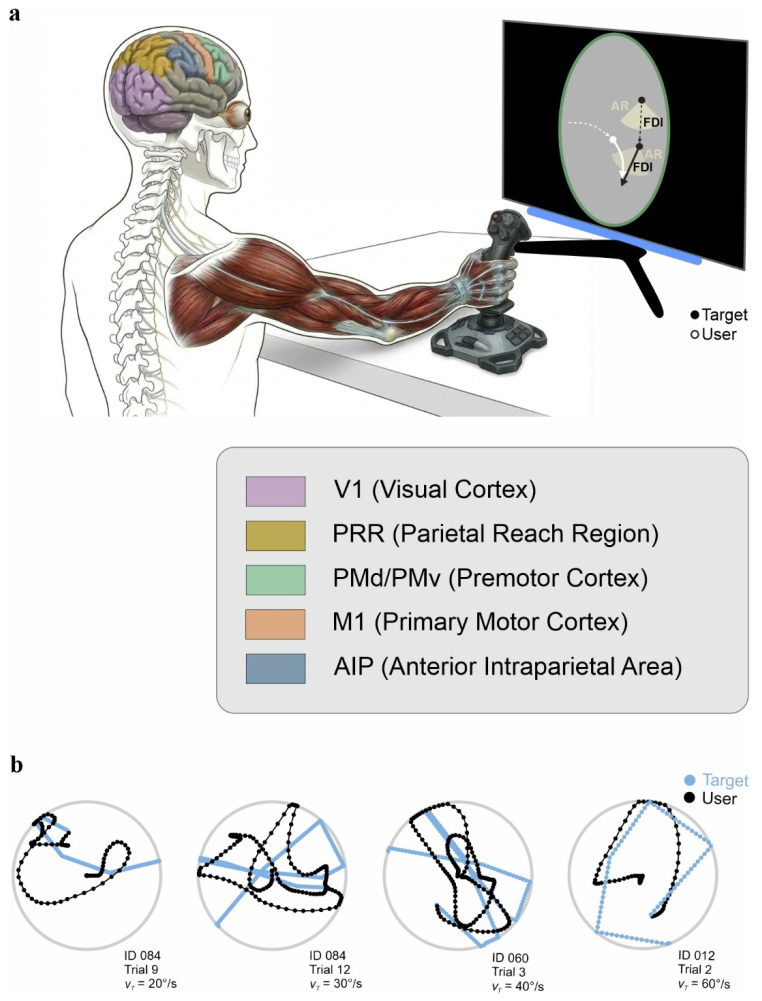
Visuomotor coordination during joystick interception (Schematic illustration). (**a**) A participant controls a joystick with the right hand during a visuomotor interception task. The diagram shows the principal cortical regions involved: visual information from V1 is transformed in parietal areas including the parietal reach region (PRR) and the anterior intraparietal area (AIP), which communicate with dorsal (PMd) and ventral (PMv) premotor cortex, respectively. These regions converge onto primary motor cortex (M1), which generates descending commands to the forearm and hand musculature. The hand is depicted with representative flexor and extensor tendons that coordinate finger movement during joystick manipulation; the thumb is controlled by specialized muscles providing independent control for precision grip. (**b**) Sample target (sky blue) and user (black) trajectories recorded during interception trials. Target trajectories are rectilinear; user trajectories exhibit more curvature reflecting joystick control dynamics. Dots indicate frame captures at matched time points. Traces in panel (**b**) adapted from Ref. [4].

The transformation of visual information into motor commands during interception relies on a distributed network spanning occipital, parietal, frontal, and subcortical regions (Figure 2). These circuits do not operate as a linear hierarchy. They form interacting loops that process motion, construct task-relevant spatial representations, and generate motor output under strict temporal constraints.

Visual motion processing begins along the dorsal stream, where signals propagate from primary visual cortex (V1) to extrastriate regions such as MT and MST. Neurons in these areas encode direction and speed, providing the basic variables required for interception [41]. These signals are not simply relayed forward. Already at this stage, motion information is shaped by contextual and behavioral demands.

A central node in this transformation is the posterior parietal cortex (PPC), a high-level associative region where sensory, motor, and cognitive signals converge. PPC integrates visual input with proprioceptive signals and efference copies of motor commands, allowing the system to maintain an updated estimate of body state and task geometry [42]. This integration supports coordinate transformations. Visual information, initially encoded in retinotopic coordinates, must be remapped into body- or effector-centered reference frames to guide action [43]. In interception, this transformation links the perceived position and motion of the target to the motor commands required to control the effector. Parietal neurons also support presaccadic attention: in a landmark finding, Duhamel et al. (1992) [44] showed that parietal neurons transiently shift their receptive fields before saccade execution, anticipating the retinal consequences of eye movements. This predictive remapping maintains a continuously updated spatial representation across saccades and provides one mechanism by which attention and motor planning are coordinated in parietal cortex.

Importantly, these transformations unfold over time. Early parietal responses provide coarse spatial estimates that can support rapid adjustments. More precise representations emerge with a delay, typically on the order of ~200 ms following a perturbation [8,45]. In parallel, anticipatory signals appear. Neuronal activity in parietal areas often reflects intended movement direction before execution, indicating that motor planning develops alongside sensory processing rather than after it [41,46]. Visuomotor transformation is therefore not an instantaneous computation but a temporally extended process shaped by both incoming input and internal estimates.

Frontal regions contribute to the specification and selection of motor plans. The frontal eye fields (FEFs) are a key region between attention, vision, and action. Neurons in FEFs encode both the location of behaviorally relevant stimuli and motor commands for saccades [47]. This encoding reflects presaccadic shifts of attention: FEF activity is tightly linked to the covert deployment of spatial attention toward the saccade target, though the relationship between oculomotor readiness and covert attention is not absolute, dissociations have been documented under specific conditions (for a review, see [48]). These representations are predominantly eye-centered, which introduces a constraint: downstream motor systems must reconcile signals expressed in different reference frames. As a result, intermediate representations are often partial or mixed, reflecting ongoing transformations rather than a single unified code.

Beyond FEFs, higher-order prefrontal regions contribute to top–down modulation of visuomotor processing. The dorsolateral prefrontal cortex (dlPFC) encodes both attentional and motor information in a flexible, task-dependent manner. Di Bello et al. (2025) [49] showed that individual dlPFC neurons can dynamically represent either the attended location or the motor target, simultaneously or at different time points, and that these mixed representations are used during the interplay between endogenous and exogenous attention. In the context of interception, the dlPFC likely contributes to the top-down regulation of attentional priority and motor selection, particularly when task demands require flexible switching between strategies.

The role of attention within this distributed network deserves explicit consideration. Attention is not a separate module but operates as a linking mechanism between sensory processing and motor control. Presaccadic attention enhances perception at the saccade target location [48], and recent evidence shows that attended stimuli serve as contextual cues that define the conditions under which sensorimotor calibration occurs [50]. In interception, this implies that the attentional state at the moment of a visuomotor decision, what is attended, and how prior selection episodes have shaped attentional priority directly modulate how sensory evidence is weighted and how motor plans are formed.

This distributed processing remains stable despite interruptions in sensory input, such as blinks or saccades. Visual perception is maintained across these discontinuities through mechanisms such as saccadic suppression and predictive remapping, in which neurons transiently encode the expected post-saccadic location of stimuli [51,52]. These processes further support the idea that visuomotor transformation relies on prospective signals rather than purely reactive mappings.

Subcortical structures are integral to this system. The superior colliculus (SC) plays a central role in orienting behavior, integrating visual, auditory, and somatosensory inputs within topographically organized maps. It contributes to both target selection and the generation of saccadic eye movements [53]. SC neurons are particularly sensitive to salient and behaviorally relevant stimuli, including looming signals that indicate potential collision [54]. Through its connections with brainstem circuits and cortical areas such as FEF, the SC enables rapid alignment of gaze with task demands.

Beyond the SC, basal ganglia circuits contribute to action selection and gating, influencing which motor plans are initiated or suppressed. These circuits modulate cortical activity based on context, reward, and task demands, shaping the selection of interceptive strategies. Brainstem pathways, in turn, implement the final motor commands, coordinating eye and head movements with short latencies.

Within this broader network, the cerebellum plays a specialized role in prediction and timing. It receives efference copies of motor commands and uses them to estimate their expected sensory consequences [23,55]. These computations are critical when feedback is delayed. In interception, cerebellar processing supports the alignment of movement with future target states before sensory confirmation becomes available. Disruption of cerebellar function leads to deficits in smooth pursuit, temporal coordination, and adaptive control, consistent with impaired predictive processing [55]. Additional contributions include short-term maintenance of motion information and regulation of limb dynamics, including impedance during ongoing action [4,11,55,56].

Other cortical regions modulate this core circuitry. Activity in dorsal anterior cingulate cortex (dACC) tracks variables such as velocity and acceleration during pursuit-like behavior, suggesting a role in monitoring ongoing performance and signaling the need for adjustment [4,11]. Recent anatomical findings further indicate direct pathways between motor cortex and cerebellum, including monosynaptic projections to cerebellar nuclei that may support rapid transitions between movement states [57]. These findings reinforce the view that visuomotor control emerges from tightly coupled, bidirectional interactions rather than feedforward processing alone.

## 4. Eye and Hand Movements as Components of Predictive Behavior

Eye and hand movements operate under distinct physical and neural constraints, and their coordination reflects these differences. The oculomotor system is fast and has low inertia. Saccades are brief ballistic events, typically lasting 40–60 ms, and once initiated they allow little online correction [58,59]. Although fatigue and adaptation can modulate their dynamics over longer timescales, individual movements remain rapid and discrete [60]. In contrast, the arm moves through a higher-dimensional mechanical space, with greater inertia and slower, continuous trajectories [4,61]. These differences produce a consistent asymmetry. Eye movements can rapidly sample the visual scene and update spatial information, whereas manual actions are slower and commit the system to a trajectory. As a result, gaze often leads the hand, especially during early phases of interception, providing spatial information that can guide subsequent motor commands. However, this lead is not fixed. Under stable conditions, gaze may anticipate future target positions; under uncertainty, it may remain closer to the target, supporting online correction. The two systems are therefore coupled but functionally distinct, contributing differently to control.

A central constraint shaping both systems is delay. Visual motion processing requires approximately 50–75 ms. Proprioceptive feedback can influence motor output within 25–60 ms, but visually guided corrections to arm movements typically emerge after 90–130 ms, with full visuomotor loops extending to 150–250 ms [62,63,64]. During this interval, a moving target can change position substantially. These delays impose a fundamental limitation: control cannot rely exclusively on current sensory input. Both eye and hand movements must incorporate information about future states or compensate for the lag.

Eye movements provide a direct window into how this compensation is achieved. During interception, smooth pursuit stabilizes the image of the moving target on the fovea, supporting precise estimation of velocity and direction [65]. When target motion changes unpredictably, pursuit lags and position error accumulates. Catch-up saccades then reposition gaze, reducing this discrepancy [66]. The alternation between pursuit and saccades reflects the limits of each mechanism: pursuit maintains continuous tracking, while saccades provide rapid correction.

Temporal coordination between eye and hand further reveals predictive structure. Gaze typically precedes manual movement by approximately 50–100 ms [5]. This lead allows early sampling of spatial information before the hand initiates movement. Under increased uncertainty, manual initiation is often delayed, while gaze continues to track the target. This asymmetry reflects differences in cost: eye movements are energetically inexpensive and easily reversible, whereas manual actions are costly and constrain future states. The system therefore uses gaze to probe and update the environment before committing the effector.

As interception progresses, oculomotor dynamics shift. Smooth pursuit gain remains relatively high, while the frequency of catch-up saccades decreases [65]. This pattern suggests a transient stabilization of gaze during the final phase of movement, reducing variability in visual input when the opportunity for correction is limited.

Gaze behavior often anticipates future events. Rather than tracking the instantaneous target position, observers frequently direct gaze toward expected future locations. This is particularly evident in structured contexts. For example, when targets rebound, gaze shifts toward the predicted post-rebound trajectory before the event occurs [3]. The precision of these anticipatory fixations depends on task parameters, indicating sensitivity to underlying dynamics.

Such behavior can be quantified using geometric metrics. The squared distance error (SDE), for instance, measures the alignment between gaze and the target’s projected future position, computed from its trajectory. Negative values indicate that gaze is directed ahead of the target, whereas positive values reflect lagging behavior [5]. Within a single trial, gaze often transitions between these regimes, indicating that predictive and reactive processes coexist and are continuously reweighted.

Experience sharpens these patterns. Skilled performers tend to generate earlier and more precise predictive saccades, often targeting the expected point of interception. Their eye–hand coordination remains stable even when visual information is degraded [67]. This suggests tighter coupling between learned environmental regularities and sensorimotor control.

From a theoretical perspective, these observations are consistent with frameworks in which the nervous system maintains internal estimates of environmental dynamics. In internal model accounts, eye movements contribute by sampling informative regions of the scene, updating these estimates [16,68]. Predictive processing and active inference formulations extend this view, interpreting saccades as actions that reduce uncertainty by minimizing prediction error [69,70]. Under this interpretation, gaze reflects the interaction between prior expectations and incoming sensory input rather than a purely stimulus-driven process [2].

Finally, physiological signals beyond movement provide additional insight into control states. Pupil diameter, for example, correlates with behavioral strategy. Larger dilations are often observed in trials associated with more effective predictive control [4]. These effects should be interpreted cautiously, as pupil size is influenced by luminance, arousal, and neuromodulatory factors. Nevertheless, they suggest that global brain states may index the engagement of predictive mechanisms [71].

## 5. Quantifying Predictive and Reactive Control

Visuomotor interception paradigms allow quantitative characterization of behavior by analyzing the geometry and timing of eye and hand trajectories. Rather than relying on qualitative descriptions, these approaches compare observed behavior with benchmark solutions derived from different control strategies. A useful decomposition separates the problem into spatial and temporal components, estimating where the target will be and when the interaction should occur. This separation is analytical rather than mechanistic; both components interact continuously during behavior.

### 5.1. Spatial Components of Predictive Control

Spatial prediction involves inferring the future trajectory of the target and aligning movement direction accordingly. This process depends strongly on early visual information. When the initial segment of a trajectory is degraded or occluded, directional accuracy declines [8]. Early motion cues therefore anchor subsequent movement, constraining the trajectory even as new information becomes available. This “front-loaded” structure resembles the use of strong initial priors: early evidence establishes a hypothesis about target motion that is only gradually updated. Similar dynamics have been described in expert interception, such as batting, where initial kinematic cues bias subsequent predictions of ball trajectory.

A central challenge is distinguishing movements that track the current target position from those that anticipate its future state. Contemporary approaches address this by placing behavior along a continuum defined by geometric benchmarks [5].

**Figure 3:**
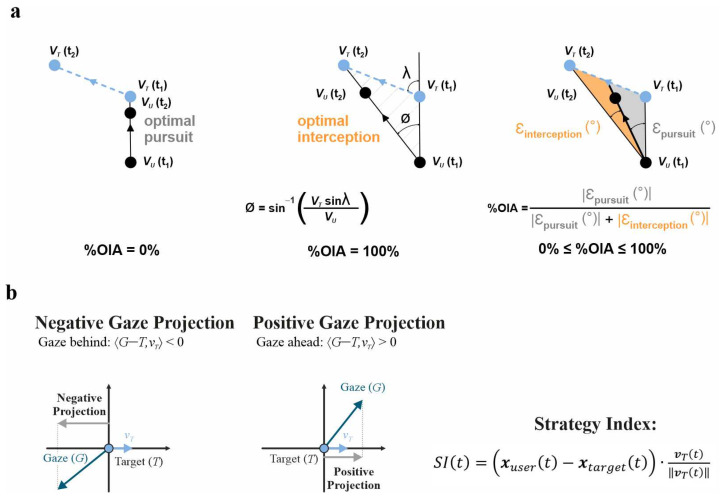
Optimal strategies to intercept a moving target (Conceptual illustration). (**a**) The target (sky blue dot) moves in a straight line at constant speed *v_T_*. The user (black dot) moves at instantaneous speed *v_U_*. Left panel: the optimal pursuit trajectory, in which the user’s velocity vector is continuously directed toward the target’s instantaneous position. Middle panel: the optimal interception trajectory, representing the shortest intercept path given the speeds of both user and target. Right panel: for each acquired frame, optimal pursuit and interception vectors are computed and compared with the participant’s observed direction. The angular error ε_pursuit_ (gray) is the deviation from the pursuit trajectory; ε_interception_ (orange) is the deviation from the interception trajectory. (**b**) Strategy index computation. The spatial offset between participant (gaze or joystick) and target positions is projected onto the target’s velocity vector. Negative values indicate reactive pursuit (spatial lag); positive values indicate predictive alignment (spatial lead). Panel (**a**) adapted from Ref. [4]. Panel (**b**) adapted from Ref. [6].

At one extreme lies manual pursuit, where the effector continuously aligns with the target’s instantaneous position, producing a lagging “tail-chase” trajectoryAt the other lies an idealized interception solution: the movement direction that minimizes time to contact under constant target velocity [4]. Observed trajectories typically fall between these extremes, and their deviation from each provides a quantitative signature of control (Figure 3).

The Optimal Interception Angle (OIA) formalizes this comparison. Given target velocity (*v_T_*), effector velocity (*v_U_*), and their spatial relation, it specifies the movement direction that would yield the earliest interception [4,11]. The OIA assumes approximately constant target velocity over the computation window and a fixed speed ratio between effector and target. Under nonlinear target motion or variable effector dynamics, the metric becomes less informative. Alignment with this solution can be expressed as a normalized index (%OIA), providing a continuous, descriptive estimate of strategy: higher values indicate greater consistency with predictive interception, whereas lower values reflect reactive tracking [4].

Distance-based metrics provide a complementary perspective. The Squared Distance Error (SDE) measures the Euclidean distance between the effector and both the target’s current position and its projected future location, computed analytically from recorded trajectories. When the effector is closer to the projected future position than to the current one, behavior exhibits a predictive signature; the opposite pattern reflects reactive control [3,5]. SDE is most diagnostic under linear or near-linear target trajectories, where the future projection is well-defined. Under abrupt direction changes or complex target dynamics, the metric can misclassify reactive corrections as apparent anticipation, particularly at coarse temporal resolution.

A related measure captures alignment along the direction of motion (Figure 3). By projecting the effector–target displacement onto the target’s velocity vector, a signed index (SI) is obtained: positive values indicate that the effector is ahead of the target, whereas negative values indicate lagging behavior. Because this metric can be computed for both gaze and hand, it reveals effector-specific differences and rapid within-trial transitions between anticipatory and feedback-driven regimes [6]. Like OIA and SDE, SI is sensitive to display geometry, effector constraints, and temporal smoothing choices, and its interpretation should be bounded by the conditions under which it was computed.

Across these measures, trajectories rarely conform to a single strategy. Early movement segments often align with predictive solutions, while later segments incorporate feedback-driven corrections as sensory evidence accumulates [5,11]. The trajectory is therefore not fixed at movement onset; it is continuously updated.

Interpretation of these metrics requires caution. Geometric similarity to an optimal solution does not imply the use of a specific computational mechanism. Predictive-looking trajectories may reflect explicit extrapolation of target motion, but they can also arise from control laws that stabilize task-relevant variables without representing future states [16,68]. Under certain conditions, reactive corrections that are rapid relative to the sampling rate can produce trajectories geometrically indistinguishable from anticipatory behavior. These measures are therefore best understood as descriptors of behavioral regimes rather than direct evidence of underlying computations.

### 5.2. Temporal Components of Predictive Control

Spatial alignment alone is insufficient for successful interception. The effector must also arrive at the correct time. Temporal prediction therefore involves estimating time-to-contact by combining target velocity with the evolving spatial gap.

Experimental perturbations in target speed reveal the flexibility of this process. When velocity changes unexpectedly, performance can be preserved by adjusting either movement timing or movement speed [8,72]. The system is not committed to a fixed temporal plan at movement onset. Instead, temporal estimates are continuously updated. When sensory input is degraded or briefly unavailable, participants compensate by shifting movement onset or modulating velocity so that arrival remains synchronized with the target [8].

Dissociations between spatial and temporal performance further support the idea that these components rely on partially independent processes. Sensitivity to directional perturbations does not strongly predict sensitivity to speed changes, and measures of feedforward precision correlate weakly with performance in tasks requiring online adaptation [8]. These results suggest that predictive control arises from interacting but dissociable subsystems. Angular perturbations tend to engage spatial tracking and external reference frames, whereas speed perturbations favor velocity-based adjustments of the effector.

Eye movements provide an additional window into temporal prediction. The latency of anticipatory saccades, the timing of fixations relative to expected events, and gaze dwell times at critical locations all carry temporal information. When saccades precede predictable events, they indicate commitment to a temporal estimate. Systematic shifts in fixation timing, in turn, reflect ongoing updating of that estimate as sensory input evolves [73,74].

## 6. Alternative and Hybrid Explanations for Interception Behavior

What kind of representation does the nervous system need to intercept a moving target? Accounts differ sharply. Internal-model and Bayesian views require the brain to encode prospective estimates of body and target state. Ecological views deny this and argue that appropriate action follows directly from optical invariants. Recent work in robotics and machine learning adds a third option: reward-driven policy learning can produce anticipatory, coordinated motor behavior without either an explicit internal model or a pre-specified perceptual heuristic. A caveat runs across all three. Descriptive regularities at the behavioral level, formal computational models, and neural implementation are separate layers of explanation. Agreement at one layer does not fix the others: a behavioral regularity consistent with a given formal account does not, by itself, identify the circuitry that produces it.

Internal-model frameworks hold that the nervous system encodes mappings between motor commands and their sensory consequences and uses them prospectively. Forward models combine the current state of the system with an efference copy of the motor command to estimate the impending sensory outcome before feedback becomes available [19]. This estimate compensates for sensorimotor delay and mechanical inertia. Deviations between predicted and observed sensory input produce prediction errors, which update subsequent actions and refine internal representations [68,75]. The term “forward model” as used here refers to the canonical formulation of sensorimotor integration [19]. Broader predictive-processing language extends these ideas into domains where the original assumptions may not fully hold, and the two should not be conflated.

Bayesian formulations cast the same process as probabilistic inference. The system holds priors over environmental dynamics and combines them with incoming sensory evidence to form posterior estimates of current and future state. Prediction errors are reliability-weighted: noisier signals influence the update less. Interception, in this view, is continuous integration of prior expectation with noisy and delayed sensory input.

**Figure 4:**
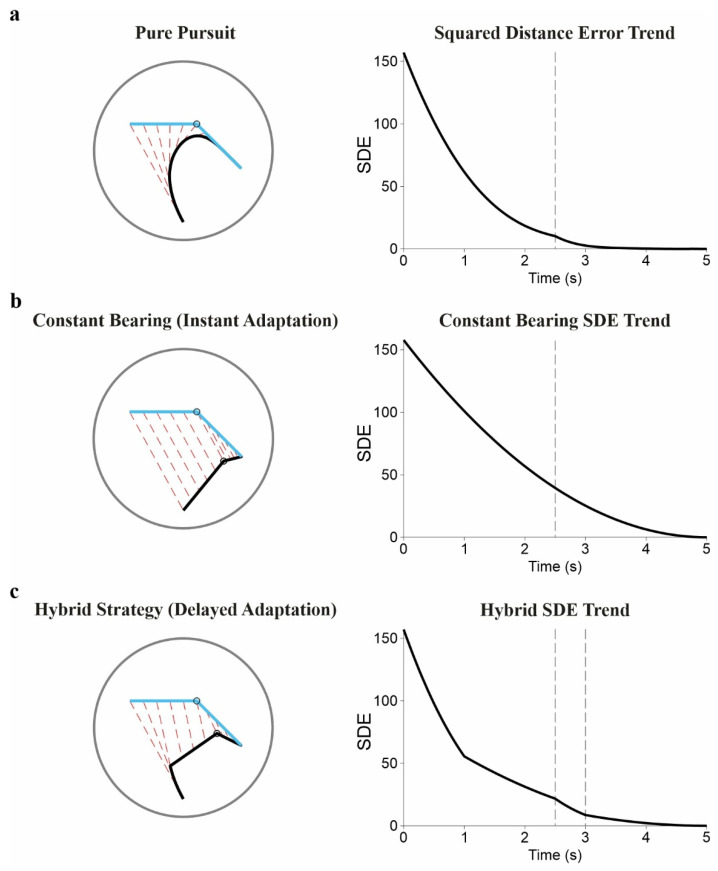
Comparison of pursuit and predictive tracking strategies in response to a sudden target maneuver (Conceptual illustration). Left column: two-dimensional spatial trajectories of a target (light blue) and user (black) within a circular arena (gray boundary). Red dashed lines connect concurrent positions at fixed time intervals to illustrate the instantaneous Euclidean distance. Right column: corresponding Squared Distance Error (SDE) over time. In all scenarios, the target moves linearly before executing a 45° direction change at t = 2.5 s (white-filled light blue circle in spatial maps; vertical dotted line in SDE plots). (**a**) Pure pursuit. The user’s velocity vector is continuously directed toward the target’s instantaneous position. The trajectory adapts immediately and smoothly to the maneuver, producing a continuous SDE profile. (**b**) Constant bearing. The user employs a predictive intercept strategy. Upon the direction change, the user instantly updates the intercept estimate (adaptation point: white-filled black circle) and adopts a new linear trajectory. (**c**) Hybrid strategy. The user begins with pure pursuit, then switches to constant-bearing prediction. Following the target’s turn, a 0.5 s sensorimotor delay (*dt*) elapses before the user updates to a new intercept vector. During this delay, the user continues the previous predictive path (second vertical dotted line in SDE plot marks the moment of adaptation). A brief description of the simulation procedures used to generate this figure is provided in the Supplemental Material.

Ecological approaches take a different route. Instead of invoking internal representations of future states, they propose that behavior is guided by information already available in the optic array. The constant-bearing strategy (CBS; Figure 4) is the canonical example: holding the bearing angle to the target constant leads to interception without explicit computation of velocity or future position [76]. Proportional navigation does something similar, using the rate of change of the line-of-sight angle as its control signal. Effective interception, on this view, arises from simple control laws grounded in organism–environment coupling.

A third option comes not from biology but from machine learning. If the referent-configuration idea shows one way to generate multi-joint movement without explicit force computation, recent robotics work pushes further. Bipedal and quadrupedal robots trained with model-free deep reinforcement learning can walk over rough terrain, recover from falls, turn, jump, run, and kick a ball, without any hand-crafted model of body or environment [77,78,79,80]. The protocol is simple in outline, if not in scale. A neural network policy acts inside a physics simulator, receives a scalar reward tied to task performance, and is updated by policy-gradient methods across millions of simulated episodes. Dynamics are randomized during training, mass, friction, actuator delays, external pushes, so that a policy that survives simulated variability transfers zero-shot to real hardware [81]. What the network learns is a mapping from state (often an action–observation history) to the next motor command. No forward or inverse model is specified beforehand, and none is required at run time. Reward-driven optimization alone is enough to produce stable, adaptive, and at times skilled motor behavior. The implication for interception is direct. If a policy trained only to maximize task reward produces anticipatory, well-coordinated action, then anticipatory trajectories, smooth online corrections, and gaze anticipation, the very patterns often read as evidence of internal prediction in humans, are not by themselves sufficient to infer an explicit internal model. The comparison has limits. Sample inefficiency, the reality gap, and the need for carefully shaped reward functions all suggest that these controllers solve the problem differently from biology. Showing that a model-free policy can produce a behavior is not the same as showing that the brain does. Virtual-rodent and musculoskeletal models trained with deep reinforcement learning have begun to reproduce features of cortical and striatal activity during movement [82,83], but the mapping from network policies to neural circuits remains tentative. How biological systems implement flexible motor control, and whether this involves internal models, learned policies, equilibrium-point dynamics, or some combination, is still open.

Most behavior falls between the internal-model and ecological positions. In locomotor interception, performance often reflects a combination of geometric strategies such as CBS and velocity-based strategies such as modified required velocity (MRV): CBS stabilizes directional relations, and MRV regulates timing by combining spatial configuration with velocity. Their relative weight depends on what information is available. Under uncertainty, behavior leans on robust geometric invariants; when sensory input is richer, velocity-sensitive adjustments take over [76]. Manual interception shows the same structure. Early movement is constrained by initial visual input and establishes coarse alignment with the target trajectory; as the movement unfolds, control becomes increasingly sensitive to incoming input, and corrections in direction and velocity compensate for accumulated error [5]. This is better described as continuous reweighting than as a switch between discrete modes. Recent work formalizes the reweighting explicitly [5,6]: gradual changes in target motion entrain both gaze and manual behavior, whereas abrupt perturbations generate large discrepancies that trigger rapid corrections whose magnitude scales with the error [84]. The distinction between prospective and feedback mechanisms is therefore temporal, not categorical. Prospective signals anticipate future states; feedback operates on delayed information to reduce emerging error. Interception lives in the interaction.

A central implication across all three frameworks is that similar behavioral patterns are not diagnostic of a single mechanism. Anticipatory trajectories, early alignment with future positions, and predictive-looking gaze patterns can arise from internal models, reward-driven policies, heuristic control laws, or direct perception–action coupling. Distinguishing between these possibilities requires more than descriptive analysis of trajectories. It requires explicit comparison between generative models that produce behavior under well-defined assumptions, ideally constrained by converging evidence from neural recordings, perturbation paradigms, and computational modeling.

One conceptual clarification belongs here. Computational prediction, motor anticipation, and the sense of agency all involve prospective processing, but they are not interchangeable. Computational prediction is the generation of an expected sensory outcome from a motor command, as in forward models. Motor anticipation is the temporal alignment of movement with a future event. The sense of agency is a subjective experience of causing an action and its effects; it varies with environmental controllability and individual traits even when the motor context is formally similar [85]. The separation matters for how interception is read. Anticipatory trajectories are evidence for motor anticipation; on their own, they say little about the form of the underlying computation and nothing about subjective agency.

## 7. Conclusions

Visuomotor interception offers a constrained setting in which multiple levels of analysis can be aligned. The task reduces behavior to a small set of measurable variables, target motion, effector dynamics, and their evolving spatial relation, while imposing strict temporal demands. Under these conditions, perception, action, and decision variables become tightly coupled: what is seen, when it is processed, and how it is acted upon all directly affect performance. This makes interception particularly suitable for examining how sensory information is transformed into action under delay.

Across behavioral, oculomotor, and analytical approaches, a consistent result emerges. Neither purely reactive nor purely predictive accounts are sufficient. Sensorimotor delays ensure that actions based only on current input are systematically outdated. At the same time, trajectories reveal continuous corrections driven by incoming sensory information, indicating that behavior is not the execution of a fixed prediction. Interception reflects the interaction of both processes. A concise summary: interception behavior is prospective but continuously corrected. Early movement reflects coarse alignment with expected target motion; later movement incorporates rapid adjustments based on updated sensory input. The balance between these components shifts with task demands and uncertainty rather than switching between discrete modes.

Prediction in this domain is also not unitary, as this review has tried to show using data from multiple groups and paradigms as well as findings from our own joystick-based interception task. Spatial and temporal components show partial dissociation. Directional alignment depends strongly on early motion information and geometric constraints, whereas timing depends on the evolving relation between velocity and distance. Experimental manipulations that selectively perturb direction or speed affect these components differently, and performance across them correlates weakly [8]. This pattern suggests that estimating where to act and when to act relies on partially distinct processes that interact during behavior but are not reducible to a single mechanism. These processes are implemented across distributed circuits. Parietal and frontal regions support the transformation of visual signals into motor plans, the cerebellum contributes predictive timing and adaptation, and subcortical structures shape selection and orienting. Peripheral mechanisms, including preparatory muscle activity and impedance modulation, further compensate for delay [27]. Eye movements provide an accessible readout of these dynamics. Anticipatory saccades and fixations often align gaze with future task-relevant locations, particularly in structured environments [3]. Importantly, these signals reflect the operation of a control system rather than a direct encoding of a single computational strategy.

What determines the relative weighting of proactive and reactive mechanisms? Task structure, sensory availability, and uncertainty are well-established factors, but motivational state likely also plays a role. Reward contingencies and task incentives can modulate both attentional deployment and motor preparation, shaping the balance between predictive and feedback-driven control. Di Bello et al. (2025) [86] showed that motivational signals sharpen the interaction between spatial attention and motor control in dorsal premotor cortex, suggesting that the engagement of predictive mechanisms is not purely perceptual or motor, it is also driven by the value structure of the task. This motivational dimension is underexplored in interception research.

A central limitation remains at the computational level. Many behavioral signatures commonly interpreted as predictive, anticipatory trajectories, early gaze shifts, alignment with future positions, are not diagnostic of a specific mechanism. Similar patterns can arise from internal models, heuristic control laws, or direct perception–action coupling. Progress therefore depends on moving beyond descriptive metrics toward explicit comparisons between generative models. This requires combining behavioral analysis with neural recordings, controlled perturbations, and model-based predictions constrained by both.

An important conceptual tool for navigating this challenge is the principle of neural degeneracy, the capacity of structurally different neural configurations or control architectures to produce similar functional outcomes [87,88]. Degeneracy implies that similar interception behaviors may arise from partially different circuit configurations, reinforcing the argument that comparable behavioral metrics are not diagnostic of a single underlying neural mechanism. Seifert et al. (2016) [89] have discussed this principle in the context of perception–action coupling, noting that degenerate solutions allow the motor system to adapt flexibly to changing task constraints. In interception, degeneracy may explain how different individuals, or even the same individual under different conditions, can achieve similar performance through distinct combinations of predictive and reactive strategies.

Within this framework, variability becomes informative. Trial-to-trial fluctuations and context-dependent adjustments reflect sensitivity to uncertainty, task structure, and learning history rather than noise alone. Expertise illustrates this principle: improvement is expressed as more efficient coordination between early trajectory formation and late-stage correction, not as rigid adherence to a single strategy.

Several directions follow. First, a clearer dissociation of spatial and temporal prediction at both behavioral and neural levels is needed. Second, quantitative frameworks that relate geometric and temporal measures to underlying control variables would improve interpretability across tasks. Third, extending interception paradigms to more naturalistic environments may reveal how these mechanisms scale under increased variability and ecological complexity. Finally, these insights extend beyond basic research. In clinical contexts, tasks that isolate spatial and temporal components of prediction may provide sensitive markers of dysfunction, particularly in conditions affecting cerebellar or visuomotor systems. In robotics and human–machine interaction, incorporating adaptive control strategies that combine prospective guidance with online correction could improve coordination with human users.

Visuomotor interception is not reduced to a single principle. It reflects the operation of a system that must act under delay, integrating prediction with ongoing sensory input. Any account, computational or biological, must accommodate that constraint.

## Data Availability

No new data were created or analyzed in this study.

## Supplementary Materials

The following supporting information can be downloaded at: https://www.mdpi.com/article/10.3390/brainsci16050435/s1, Supplemental Material for Mechanisms of Visuomotor Interception.

## Abbreviations

dACC	Dorsal anterior cingulate cortex
dlPFC	Dorsolateral prefrontal cortex
FEF	Frontal eye fields
GTD	Gaze–target distance
GUD	Gaze–user distance
MT	Middle temporal visual area
MST	Medial superior temporal area
OFC	Optimal feedback control
OIA	Optimal interception angle
PETH	Peri-event time histogram
PPC	Posterior parietal cortex
SC	Superior colliculus
SDE	Squared distance error
SI	Signed index
TTC	Time-to-contact
UCM	Uncontrolled manifold
V1	Primary visual cortex
*v_G_*	Gaze Velocity
*v_T_*	Target Speed
