# Supplementary material for: Mechanisms of Visuomotor Interception
Source: Brain Sci. 2026 Apr 22;16(5):435. doi: 10.3390/brainsci16050435 (PMC13205071; doi:10.3390/brainsci16050435)
Supplement: Supplementary file 1 [file brainsci-16-00435-s001.zip › Supplementary_material_v1.pdf]

brain sciences

MDPI

Review

Supplemental Material for Mechanisms of Visuomotor Interception

Inmaculada Márquez [1,2,3] and Mario Treviño [1,*]

[1] Laboratorio de Plasticidad Cortical y Aprendizaje Perceptual, Instituto de Neurociencias, Universidad de Guadalajara, Francisco de Quevedo 180, Arcos Vallarta, Guadalajara 44130, Jalisco, Mexico; maria.marquez@academicos.udg.mx
[2] Departamento de Psicología, Centro Universitario de la Ciénega, Universidad de Guadalajara, Ocotlán 47810, Jalisco, Mexico
[3] Laboratorio de Neurofisiología, Departamento de Bioingeniería Traslacional, Centro Universitario de Ciencias Exactas e Ingenierías, Guadalajara 44430, Jalisco, Mexico
* Correspondence: mario.trevino@academicos.udg.mx

2. Materials and Methods

2.1. Simulation of Adaptive Pursuit Strategies

To evaluate differences in sensorimotor adaptation during dynamic tracking, a two-dimensional kinematic simulation was developed in MATLAB (MathWorks, Natick, MA). The simulation modeled a 5-second trial with a discrete time step of 0.05 s. The target was programmed to move along a linear trajectory at a constant velocity for the first 2.5 s, after which it executed a sudden 45-degree direction change. We simulated a user tracking the target from a fixed starting position using three distinct control strategies:

Pure Pursuit: The user's velocity vector was continuously updated to point directly at the target's instantaneous spatial coordinates. This model assumed continuous, zero-delay sensorimotor feedback.

Constant Bearing (Predictive): The user calculated a linear intercept vector based on the target's initial velocity. Upon the target's direction change, the user's predictive model updated instantaneously (0 s delay) to a new intercept trajectory based on the target's updated velocity.

Hybrid Strategy: The user initiated tracking using pure pursuit to close the initial spatial gap, then transitioned to a constant bearing predictive strategy. To model human reaction limits, a 0.5 s sensorimotor perception delay was introduced. Following the target's maneuver, the user continued the obsolete path for 0.5 s before recalculating the correct intercept vector.

Performance for all three strategies was quantified continuously by calculating the Squared Distance Error (SDE) between the user and the target at each time step.

Academic Editor: Aasef G. Shaikh

Received: 27 March 2026
Revised: 19 April 2026
Accepted: 21 April 2026
Published: 22 April 2026

Copyright: © 2026 by the authors. Submitted for possible open access publication under the terms and conditions of the Creative Commons Attribution (CC BY) license.

2.2. Simulation of Optic Flow Braking (τ Theory)

To illustrate the principles of Time-to-Contact (τ) theory, a second MATLAB simulation modeled a one-dimensional braking approach toward a stationary target. The simulation used a discrete time step of 0.02 s. The actor began at an initial distance of 100 m with an approach velocity of 20 m/s. The target was assigned a physical width of 2 m to allow for the calculation of the visual angle (θ) subtended on the actor's retina, simulating the optical "looming" effect. The optical variable τ was defined as the inverse of the relative rate of retinal expansion, which mathematically simplifies to the instantaneous distance divided by velocity (x/v). The simulation evaluated the actor's trajectory based on the temporal derivative of tau ($\dot{\tau}$) under three constant-deceleration conditions:

Marginal/Ideal Braking: A deceleration of 2.0 m/s² was applied, maintaining the critical threshold of $\dot{\tau}$ = -0.5. This resulted in the actor's velocity reaching zero at the exact moment the distance reached zero.

Safe Braking: A higher deceleration of 2.5 m/s² was applied, keeping the rate of change above the critical threshold ($\dot{\tau}$ > -0.5), resulting in the actor safely stopping short of the target.

Hard Braking (Collision): A lower deceleration of 1.5 m/s² was applied, causing the rate of change to drop below the threshold ($\dot{\tau}$ < -0.5), resulting in the actor colliding with the target before velocity reached zero.
